# Changes in the Nervous Tissues Due to Uremia

**Published:** 1898-02

**Authors:** 


### CHANGES IN THE NERVOUS TISSUES DUE TO UREMIA.

Two Italian observers, Acquisto and Pasateri, in a number of experiments made upon animals, report in the *Revista di Patologia nervose e mentale*, Vol. II. No. 10, a number of histological and anatomical changes.

The animals mainly experimented upon were dogs. Artificial uremia was induced in them by ligation of the ureters, the dogs dying in from sixty-eight to ninety-six hours. The nervous tissues were variously fixed and stained by the methods of Golgi and Nissi. In the cortex of the brain no changes were found in the large ganglion-cells, the axis cylinder-processes, the neuroglia, the pericellular spaces, nor the perivascular spaces. The cells showing the greatest amount of change were the smaller ganglion-cells and their dendrites, the latter showing conditions of varicose atrophy in some or all of their branches. The ganglion-cells were chromatolytic, the chromophylic substance being found in the form of round granules, generally in the center of the cell, sometimes near the dendrites. The perinuclear zone was usually clear and homogeneous. The large cells of the cord were similarly affected.—*American Medico-Surgical Bulletin.*

